# Implications of noncoding RNAs for cancer therapy: Are we aiming at the right targets?

**DOI:** 10.37349/etat.2025.1002286

**Published:** 2025-01-16

**Authors:** Amil Shah

**Affiliations:** European University Cyprus, Cyprus; Department of Medicine, University of British Columbia, Vancouver, BC V5Z 1M9, Canada

**Keywords:** Tumorigenesis, noncoding RNA, RNA regulatory network, cancer initiation, targeted therapy, nucleic acid-based therapy

## Abstract

The discovery of oncogenes and tumor suppressor genes led to a better understanding of tumorigenesis, and prompted the development of molecularly targeted therapy. Over the past 30 years, many new drugs, which are primarily aimed at activated oncogenic proteins in signal transduction pathways involved in cell proliferation and survival, have been introduced in the clinic. Despite its rational design, the overall efficacy of targeted therapy has been modest. Recently, the noncoding RNAs (ncRNAs) have emerged as key regulators of important cellular processes in addition to the known regulatory proteins. It now appears that dual epigenetic regulatory systems exist in higher eukaryotic cells: a ncRNA network that governs essential cell functions, like cell fate decision and maintenance of homeostasis, and a protein-based system that presides over core physiological processes, like cell division and genomic maintenance. Modifications of the ncRNA network due to altered ncRNAs can cause the cell to shift towards to neoplastic phenotype; this is cancer initiation. Mutations in the well-known cancer driver genes provide the incipient cancer cell with a selective growth advantage and fuel its consequent clonal expansion. Because of the crucial role of the altered ncRNAs in tumorigenesis, targeting them may be a reasonable therapeutic strategy.

## Background

The prevailing somatic mutation theory of tumorigenesis states that a cancer starts with cells that acquire specific mutations, which confer on them a selective growth advantage. As a result, large descendant cell populations are spawned within the primary tumor. Further mutations lead to the hallmarks of cancer: autonomous cell proliferation, evasion of growth suppression, resistance to apoptosis, recruitment of new blood vessels, avoidance of immune destruction, a switch to glycolytic metabolism, and, finally, metastasis to distant anatomical sites in the body [1]. The discovery of oncogenes and tumor suppressor genes in the 1970s supported this model. Indeed, the well-known adenoma to carcinoma sequence in colon cancer nicely illustrates the gradual, stepwise transition from normalcy to malignancy. A mechanistic concept to explain the progression to malignancy is that the cell’s elaborate signaling system is subverted [2]. According to this, oncogenes and tumor suppressor genes encode numerous signaling molecules that serve as nodes and branches in signaling pathways that interact with one another to form overlapping circuits. These are divided into distinct subcircuits, each of which supports a discrete biological function. The individual subcircuits are highly interconnected with robust crosstalk among them. In cancer, mutations rewire the cellular circuitry, giving rise to its hallmark features.

The notion that tumorigenesis is driven by specific mutations makes targeting them an attractive proposition as a treatment modality. Over the past 30 years, molecularly targeted therapy has become another routine treatment for cancer. This largely consists of small molecule tyrosine kinase inhibitors and monoclonal antibodies, which inhibit signal transduction pathways involved in cell growth, proliferation, and survival [3]. Many protein kinases are dysregulated in cancer due to activation by gain-of-function mutation, gene amplification and chromosomal rearrangement, and they are, therefore, prime candidates for targeted therapy. A second class of targeted therapy is monoclonal antibodies, which are directed to extracellular ligands like vascular endothelial growth factor (VEGF), membrane receptors like human epidermal growth factor receptor 2 (HER-2) or epidermal growth factor receptor (EGFR), and membrane-bound proteins like B-lymphocyte antigen CD20 (CD20). More recently, immune checkpoint inhibitors, which directly or indirectly activate host antitumor immunity, have been introduced; these include antibodies against programmed cell death protein 1 (PD-1), programmed death-ligand 1 (PD-L1), and cytotoxic T-lymphocyte-associated protein 4 (CTLA-4). Targeted therapy is successful in some uncommon cancers, such as the tyrosine kinase inhibitors of the Bcr-Abl1 chimeric tyrosine kinase that results from reciprocal translocation between the *BCR* (break-point cluster region) gene on chromosome 22 and the Abelson (*ABL1*) gene on chromosome 9 in chronic myelogenous leukemia. Overall, however, despite the rational design of these drugs, their efficacy is modest, and their optimal use in the overall management of cancer patients remains under study [4]. Further, it was anticipated that blockage of specific mutant proteins would be less toxic than traditional chemotherapy, which affects both cancerous and healthy tissues. Unfortunately, the toxicity of targeted therapy is not inconsequential due to the unexpected cross-reactivity with normal cells. Moreover, the emergence of drug resistance with subsequent cancer relapse is a major obstacle to success.

It is worthwhile to take stock of the overall impact of current targeted therapy. In this regard, some questions have recently been raised about the exact role of oncogenes and tumor suppressor genes in cancer development [5, 6]. A surprising discovery is the association of the cancer genes with benign conditions like nevi or rheumatoid arthritis. Even more unexpected is that cancer genes are prevalent in healthy aging tissues, in which a cancer seldom develops. Further, the spectrum of oncogene and tumor suppressor gene mutations is not consistent even among cancers of the same type. It is also now recognized that there are innumerable mutations due to kataegis, chromothripsis, and chromoplexy in almost all cancers [7]. This brings up the contention that of the myriad mutations in a typical cancer, only four or five putatively drive tumorigenesis, while more than 99% are passenger changes that do not contribute to cancer development [2, 7]. Moreover, the mutations responsible for metastasis, typically a late event in the course of most cancers, are, in fact, present relatively early during tumorigenesis, where they also provide a proliferative advantage to the incipient tumor cells [8]. These observations highlight a striking conceptual inconsistency: mutant cancer genes specify multiple traits that are seemingly not in line with the stepwise progression of cancer. Indeed, it appears that the effects of the mutations may depend on the cellular environment, and different paths can lead to a specific type of cancer [9]. It is hard to reconcile these discrepancies with the sequential acquisition of a few driver mutations. Recently, the proposition was put forward that a cancer is, in fact, initiated by multiple mutations, which reprogram the cell’s epigenetic program, causing a large shift towards a neoplastic phenotype [10].

## The rise of noncoding RNAs

A pertinent discovery is that 98% of the human genome does not encode proteins, but are, nevertheless, transcriptionally active and give rise to a wide range of noncoding RNAs (ncRNAs) [11]. These originate in the introns of protein-coding genes, the intergenic regions, and other transcripts of DNA segments that do not encode proteins. Several independent lines of evidence point to ncRNAs as an integral part of the cell’s epigenetic apparatus with complex regulatory functions [12]. They coordinate the massive flow of genetic information responsible for the organization of the sophisticated biological activities of the eukaryotic cell. Indeed, it appears that the proportion of the genome transcribed into ncRNAs is more reflective of the complexity of an organism than the number of its protein-coding genes [13].

ncRNAs are divided into two classes. First, housekeeping ncRNAs, which are constitutively expressed in relatively stable concentrations across different cell types, subserve basic cell functions. The second class is the regulatory ncRNAs, which comprise short ncRNAs and long ncRNAs [14]. They function in different cellular compartments and participate in a variety of processes, including transcriptional regulation, RNA processing and modification, and messenger RNA stability and translation. Their expression is dynamic in response to specific cellular conditions. Table 1 lists some of the regulatory ncRNAs and their diverse, overlapping functions.

**Table 1:** ncRNAs: types, average nucleotide (nt) lengths, and key functions

**Class**		**Type**	**Length (nt)**	**Function**
Housekeeping ncRNAs		Ribosomal RNA (rRNA)	> 1,500	Protein synthesis
		Transfer RNA (tRNA)	76–90	Protein synthesis
		Small nuclear RNA (snRNA)	100–300	RNA processing
		Small nucleolar RNA (snoRNA)	60–200	Pre-mRNA processing
Regulatory ncRNAs	Short ncRNAs (< 200 nt)	MicroRNA (miRNA)	18–22	Protein translation regulation
		Small interfering RNA (siRNA)	20–25	Post-translation mRNA regulation; antiviral defense
		Piwi-interacting RNA (piRNA)	26–31	Silencing of transposable elements
	Long ncRNAs (> 200 nt)	Long ncRNAs (lncRNAs)	~ 1 kb	Spatiotemporal gene expression (cell differentiation); modification of 3D chromatin architecture; protein scaffolding; miRNA sponging
		Circular RNA (circRNA)	100–999	miRNA sponging; protein scaffolding; gene expression (cell/tissue development)

3D: three-dimensional; ncRNAs: noncoding RNAs

Although knowledge of the full range of molecular functions of the heterogenous group of ncRNAs continues to be gathered, the evidence points to important regulatory roles in gene expression during development and in maintenance of cellular homeostasis. The targets of ncRNA regulators are other RNAs as well as DNA, with which they engage through sequence-specific signals, but they also interact with regulatory proteins. The rich connectivity among the cell’s biomolecules gives rise to networks within each tissue type that determine which genes are turned on, and when.

It appears that two intertwined systems, a ncRNA system and a protein-based system, exist in the cell for the control of gene expression [15]. ncRNAs function upstream of master transcription factors as well as feed information into the cell’s transcriptional machinery to fine tune its activity. A major way by which this control is achieved is through enhancers, which carry the regulatory instructions for spatiotemporal gene expression. These instructions are communicated to the cognate gene promoters through the dynamic interactions of the various regulatory factors that include *cis-*regulatory elements, *trans*-acting transcription factors and signaling molecules [16]. An integral component of enhancers is enhancer-derived long ncRNAs (elncRNAs), which are transcribed from the enhancers themselves. elncRNAs modify the three-dimensional chromatin architecture of the DNA loops within specific topologically associating domains (TADs) that serve as transcriptional hubs for cell development. Of note, there are an estimated 400,000 putative enhancers in the human genome, far in excess of the total number of genes. It follows that an individual gene can be regulated by many enhancers; in turn, one enhancer can regulate several genes. This built-in redundancy is a common theme in the ncRNA network.

## The role of ncRNAs in tumorigenesis

Most studies of the cancer genome have focused on oncogenes and tumor suppressor genes, and the mutations that modulate tumor progression. More recently, as the central role of ncRNAs in cellular processes has been become increasingly recognized, they have been linked to cancer development [17]. A significant determinant of cancer is the multitude of different genetic variants present in the human genome [18]. Unlike mutations of oncogenes and tumor suppressor genes, these variants are only marginally functional or positively selected. About 90% of the genetic variants are single nucleotide variants (SNVs), which are the major form of genetic polymorphisms. Of the > 14 million polymorphisms in the human genome, 38% are in protein-coding genes, while the majority (62%) resides in intergenic regions [19]. Moreover, within the genes, most of the variants are in introns with small fractions found within the coding regions, 5’ UTRs and 3’ UTRs. Thus, most of genetic polymorphisms fall within the noncoding regions where ncRNA is synthesized.

SNVs affect the function of ncRNAs in various ways [17]. By modifying protein-binding motifs or secondary structures, like hairpin loops, the interaction of ncRNAs with their target molecules is altered. Additionally, SNVs in the 5’ UTR affect translation, while those in 3’ UTR influence post-transcriptional gene expression by disrupting RNA-RNA or RNA-protein interactions. SNVs can also generate variant noncoding regulatory sequences that affect downstream signaling of oncogenic proteins, demonstrating the functional connectivity between ncRNAs and oncogenic proteins. For example, long ncRNAs (lncRNAs) can act as microRNA (miRNA) sponges and regulate protein-coding driver gene expression in prostate cancer [20]. The regulation is not straightforward; an individual driver gene can be regulated by multiple lncRNAs, and one lncRNA can coregulate many driver genes. Further, certain miRNAs and lncRNAs regulate cell-cycle proteins, like cyclin-dependent kinases and their associated cyclins, at the transcriptional and translational levels, and, therefore, control their expression at different cell-cycle phases. Alterations in these ncRNAs can disrupt cell-cycle regulation and are linked to tumorigenesis [21]. In addition, chromosomal translocations and gene amplifications, which are common findings in cancer, contribute to cancer by affecting ncRNA integrity or by increasing the number of copies of ncRNA. For example, chromosomal translocation can bring together two complementary repetitive intronic sequences (Alu elements), favoring back-splicing events to produce aberrant circular RNA (circRNAs), called fusion circular RNAs or f-circRNAs; this occurs in about 50% of translocations in cancer [22]. Normally, circRNAs control gene expression by sponging miRNAs, but f-circRNAs can lead to increased cell proliferation. Specific circRNAs also maintain the stemness and pluripotency of both embryonic and adult stem cells as well as determine stem cell differentiation and tissue development. Deregulation of circRNA expression results in an imbalance between self-renewal and differentiation, and, thus, can contribute to tumorigenesis. Finally, altered adenosine-to-inosine (A-to-I) RNA editing in 3’ UTR perturbs miRNA-mediated regulation of some cancer-associated genes [23]. The 3’ UTR of RNA often contains alternative polyadenylation signals, which allow the production of isoforms with multiple 3’ UTRs derived from a single gene. The generation of alternative switches for the same transcript allows the fine-tuning of expression of certain genes in specific tissues. However, 3’ UTR shortening in cancer cells alters miRNA targeting in ncRNA networks by deleting any regulatory components, such as miRNAs, that it may contain. This loss of miRNAs can lead to oncogene activation.

In summary, ncRNAs are a diverse group of RNA molecules that have distinct regulatory mechanisms, functional domains and different forms of biogenesis compared with other gene transcripts. Aberrant expression, mutations and SNVs of ncRNAs are associated with tumorigenesis. While ncRNAs can deregulate oncogenic and tumor suppressor gene pathways via different mechanisms, it also appears that many cancer-related ncRNAs drive the transformed phenotype of the cancer cell by reshuffling the dynamic nature of their interactions.

## Cancer as a disruption of cell development

Among the diverse functions of ncRNAs, their role in cell lineage pathways is pivotal in enabling the creation of the full complement of about 250 cell types, totaling 4 × 10^13^ cells in the adult human [24]. The execution of the cell developmental program requires sophisticated genome-level regulation and accurate spatiotemporal gene expression to coordinate specific cell functions and cell-cell interactions. Not surprisingly, therefore, the genes involved in this process are more tightly controlled than other groups of genes. There are two prominent features of this regulation. First, various ncRNAs, abundant transcription factors and enhancers typically participate in frequent chromatin-chromatin interactions. Second, the process is dynamic with feedback loops that provide robustness, ensuring that epigenetic or environmental perturbation has a minimal effect on the phenotype [25, 26].

The dynamic nature of the molecular interactions involved in the regulation the genome has led to the recognition of gene expression as a probabilistic process rather than a deterministic one [26]. An important consequence of this behavior is the emergence of a self-organizing system that exhibits a tendency to settle down into an overall state at which the system is at equilibrium. This defines an “attractor” state, which in the genome corresponds to the gene expression profile of a distinct cell type. The concept of attractors is particularly relevant to cell differentiation, in which there is a transition between attractors as gene expression programs are modulated to generate new, stable cellular states. The high degree of genomic plasticity leads to stochastic heterogeneity among cells in a population, and individual cells may respond differently to a stimulus by entering the differential pathway more quickly, depending on their gene expression program.

It is important to note that in line with probabilistic and combinatorial nature of the dynamic interactions of regulatory molecules, which directly or indirectly influence the expression of one another, that a theoretically large number of gene-expression programs or phenotypes are generated. In this large universe of possibilities, cells with potentially different biological fitness arise. Over evolutionary time, however, those genetic programs that lead to functional cell states have been selected from these myriad possibilities. The result is a genomic landscape which is “canalized” and streamlined, and in which pathways to less fit attractors are bypassed and are not generally accessible [27]. However, mutations can reshape the contours of the landscape and enable state transitions, allowing cells to drift from regular differentiation pathways into unused attractor states, among which are gene-expression configurations that encode a neoplastic phenotype [28].

This view of gene regulation dynamics suggests that a functional error in cell development is the initiating cause of a cancer [29]. In other words, a perturbation of the ncRNA regulatory network is responsible for the developmental miscue that triggers tumorigenesis. This perspective has important implications about how we think about the origin of cancer. It differs in important ways from the somatic mutation theory, which asserts that of the large number of mutations identified in a cancer, a few drive the cancer process [2]. In contrast, regulatory ncRNAs operate more subtly. First, the built-in redundancy in the system makes it robust and provides remarkable buffering against variable or perturbed inputs, such as loss or dysfunction of individual regulatory elements. Second, individual components of the ncRNA circuitry exert a small overall effect, unlike cancer gene mutations whose impact is typically manifest.

Taken together, the interwoven, redundant nature of the ncRNA network and the contained, minimal fall-out from alterations of its individual elements are fitting properties of a master regulatory system, but when there are multiple changes in the ncRNAs, their circuitries may be rewired. As a consequence, the gene-expression configuration, which accounts for the distinctiveness of the cell, is modified. Ultimately, a tipping point is reached and the cell phenotype changes. Cells are programmed to undergo apoptosis in response to significant genomic changes, and this is the likely fate for most. However, when the damage is sublethal, the cell drifts towards a neoplastic phenotype; this marks the onset of tumorigenesis or cancer initiation [10]. When mutations of the oncogenes and tumor suppressor genes, which subserve major physiological processes like the cell cycle and apoptotic pathways, occur in the milieu of the initiated cancer cell, they provide it with a selective growth advantage.

Finally, it noteworthy that disruption of ncRNA functions is associated with various benign diseases, including neural, muscular, cardiovascular, adipose, hematopoietic and immune disorders [12, 17]. However, unlike cancer, where ncRNA alterations lead to a fundamental phenotypic shift by rewiring the cell’s central regulatory circuity, the ncRNAs involved in benign conditions are typically linked to critical biochemical pathways or specific signaling hubs within the ncRNA network.

## The path forward

The proposition that dysregulation of the ncRNA network is the underlying cause of cancer means that ncRNAs ultimately dictate its epigenetic blueprint. Because the ncRNAs associated with different cancer types have expression patterns that are tumor specific, they are valid therapeutic targets. Nucleic acid-based therapeutics are a novel class of drugs with the potential to treat cancer at the genetic level in contrast to traditional protein-based targeted therapy (small molecule drugs or antibodies). Nucleic acid-based therapeutics include RNA molecules, such as antisense oligonucleotides, small interfering RNAs (siRNAs), short hairpin RNAs (shRNAs), anti-miRNAs, miRNA mimics, miRNA sponges, and therapeutic circRNAs, which directly target any ncRNA of interest through complementary base-pairing, to modulate its expression and function [12]. Rapid advances continue to be made in delivery platforms for nucleic acid-based therapeutics, and several drugs are now in clinical trials for cancer [30].

It is also important to recognize that each new nucleic acid-based drug brings with it unknown or unexpected side effects, such as cellular off-target effects, systemic mistargeting or immune activation [31]. An additional challenge is posed by the wide-reaching cellular effects of the complex ncRNA regulatory network. The patterns of connectivity of its nodes determine not only the network’s output, but its vulnerability to perturbations [26]. Hence, a minor modulation by a therapeutic agent could have unpredictable and unintended downstream consequences. Moreover, multiple nodes are likely dysregulated during tumorigenesis; this makes containment of aberrant network signaling a daunting task. A high level of vigilance regarding patient safety is, therefore, required as these new drugs are introduced into the clinic.

An improved understanding of molecular functions of the various ncRNAs is an essential first step to understand their regulatory roles in the multi-level complexity of the human genome. Of tantamount importance is elucidating the dynamic links and balances among the many ncRNA species in cell processes and cell states. Progress in this area will require large-scale genome studies and high quality experimental functional validation coupled with the analytical power of bioinformatics to decipher their organization and dynamics.

## Abbreviations

CD20: B-lymphocyte antigen CD20
circRNA: circular RNA
EGFR: epidermal growth factor receptor
HER-2: human epidermal growth factor receptor 2
lncRNAs: long noncoding RNAs
miRNA: microRNA
ncRNAs: noncoding RNAs
SNVs: single nucleotide variants
VEGF: vascular endothelial growth factor

## Declarations

### Copyright

© The Author(s) 2025.
